# Poly[[μ_2_-aqua-tetraaquadi-μ_3_-malonato-nickel(II)strontium(II)] dihydrate]

**DOI:** 10.1107/S1600536810049779

**Published:** 2010-12-04

**Authors:** Ming-Lin Guo, Long Liu, Cong-Cong Lu

**Affiliations:** a School of Environment and Chemical Engineering and Key Laboratory of Hollow Fiber Membrane Materials & Membrane Processes, Tianjin Polytechnic University, Tianjin 300160, People’s Republic of China

## Abstract

The unit-cell parameters for the title mixed-metal coordination polymer, {[NiSr(C_3_H_2_O_4_)_2_(H_2_O)_5_]·2H_2_O}_*n*_, which is isostructural with its Co-containing analogue, were reported previously [Gil de Muro *et al.* (1999 ▶). *Eur. J. Inorg. Chem.* pp. 935–943]; the full crystal structure including a description of the hydrogen bonding is reported here. The Sr^2+^ ion is bonded to five O atoms from three different malonate dianions and four water mol­ecules, displaying a distorted tricapped trigonal–prismatic coordination geometry. Two malonate dianions, two water mol­ecules and one Ni^2+^ ion build up a dianionic [Ni(C_3_H_2_O_4_)_2_(H_2_O)_2_]^2−^ unit incorporating a slightly distorted NiO_6_ octa­hedron, which coordinates to three nearby Sr^2+^ ions. This arrangement creates a metal-organic framework having a 20-membered ring with four Ni and six Sr atoms lying in the *bc* plane. The coordinated and uncoordinated water mol­ecules are responsible for the formation of two *D*5 hydrogen-bonded water chains within the 20-membered ring and they are linked into an *R*4 water cluster *via* two bifurcated O—H⋯(O,O) links.

## Related literature

For the cobalt-containing analogue of the title compound and the previous unit-cell determination, see: Gil de Muro *et al.* (1999 ▶). For a related structure, see: Gil de Muro *et al.* (2000 ▶). For hydrogen-bonded water clusters, see: Infantes & Motherwell (2002 ▶). For graph-set notation, see: Bernstein *et al.* (1995 ▶).
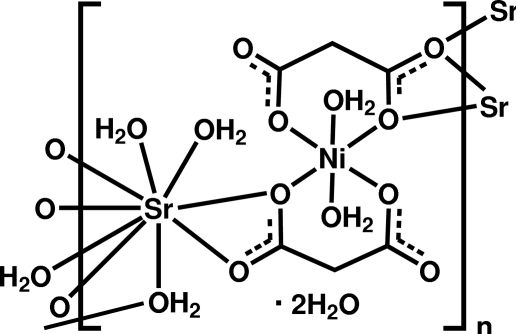


## Experimental

### 

#### Crystal data

[NiSr(C_3_H_2_O_4_)_2_(H_2_O)_5_]·2H_2_O
*M* *_r_* = 476.53
Monoclinic, 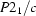

*a* = 6.7745 (14) Å
*b* = 14.220 (3) Å
*c* = 15.629 (3) Å
β = 101.10 (3)°
*V* = 1477.4 (5) Å^3^
*Z* = 4
Mo *K*α radiation
μ = 4.97 mm^−1^
*T* = 294 K
0.12 × 0.06 × 0.04 mm

#### Data collection

Rigaku Saturn CCD area-detector diffractometer
Absorption correction: multi-scan (*CrystalClear*; Rigaku/MSC, 2005 ▶) *T*_min_ = 0.548, *T*_max_ = 0.712
9983 measured reflections
2609 independent reflections
2235 reflections with *I* > 2σ(*I*)
*R*_int_ = 0.045

#### Refinement

*R*[*F* ^2^ > 2σ(*F* ^2^)] = 0.035
*wR*(*F* ^2^) = 0.098
*S* = 1.05
2609 reflections
208 parameters
H-atom parameters constrained
Δρ_max_ = 0.78 e Å^−3^
Δρ_min_ = −0.59 e Å^−3^

### 

Data collection: *CrystalClear* (Rigaku/MSC, 2005 ▶); cell refinement: *CrystalClear*; data reduction: *CrystalClear*; program(s) used to solve structure: *SHELXS97* (Sheldrick, 2008 ▶); program(s) used to refine structure: *SHELXL97* (Sheldrick, 2008 ▶); molecular graphics: *SHELXTL* (Sheldrick, 2008 ▶); software used to prepare material for publication: *SHELXTL*.

## Supplementary Material

Crystal structure: contains datablocks I, global. DOI: 10.1107/S1600536810049779/hb5748sup1.cif

Structure factors: contains datablocks I. DOI: 10.1107/S1600536810049779/hb5748Isup2.hkl

Additional supplementary materials:  crystallographic information; 3D view; checkCIF report

## Figures and Tables

**Table 1:** Selected bond lengths (Å)

Sr1—O11	2.556 (2)
Sr1—O12	2.574 (3)
Sr1—O2^i^	2.581 (3)
Sr1—O6^ii^	2.598 (3)
Sr1—O13	2.618 (3)
Sr1—O2	2.660 (3)
Sr1—O13^iii^	2.688 (2)
Sr1—O1	2.751 (3)
Sr1—O5^ii^	2.816 (3)
Ni1—O4	2.020 (3)
Ni1—O7	2.024 (3)
Ni1—O5	2.026 (2)
Ni1—O1	2.032 (3)
Ni1—O9	2.038 (3)
Ni1—O10	2.064 (3)

Symmetry codes: (i) 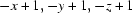; (ii) 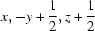; (iii) 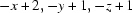.

**Table 2:** Hydrogen-bond geometry (Å, °)

*D*—H⋯*A*	*D*—H	H⋯*A*	*D*⋯*A*	*D*—H⋯*A*
O15—H15*A*⋯O14^iv^	0.84	2.26	2.997 (5)	148
O15—H15*A*⋯O14	0.84	2.33	2.867 (5)	123
O15—H15*B*⋯O3^v^	0.85	2.29	2.881 (7)	127
O14—H14*B*⋯O8^vi^	0.86	2.21	3.072 (5)	176
O14—H14*A*⋯O10^ii^	0.86	2.14	2.936 (4)	152
O13—H13*B*⋯O11^i^	0.85	1.93	2.728 (4)	155
O13—H13*A*⋯O7^iii^	0.85	2.01	2.836 (4)	163
O12—H12*B*⋯O7	0.85	2.42	3.080 (4)	135
O12—H12*B*⋯O9	0.85	2.36	3.094 (5)	144
O12—H12*A*⋯O3^vii^	0.85	1.94	2.772 (4)	166
O11—H11*B*⋯O8^vi^	0.85	1.83	2.681 (4)	176
O11—H11*A*⋯O4^ii^	0.85	1.89	2.727 (4)	172
O10—H10*B*⋯O6^viii^	0.85	1.91	2.728 (4)	162
O10—H10*A*⋯O3^ix^	0.85	1.86	2.714 (4)	178
O9—H9*B*⋯O15	0.85	1.84	2.663 (5)	162
O9—H9*A*⋯O8^vi^	0.84	1.81	2.652 (4)	173

Symmetry codes: (i) 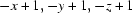; (ii) 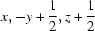; (iii) 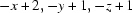; (iv) 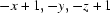; (v) 
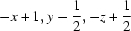
; (vi) 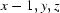; (vii) 
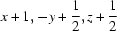
; (viii) 
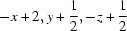
; (ix) 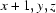.

**Fig. 1.**
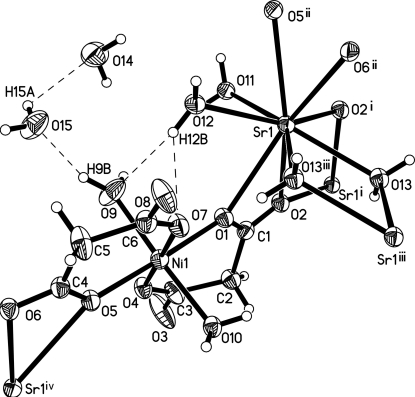
A view of the structure of (I), showing the coordination environment for Sr and Ni atoms; displacement ellipsoids were drawn at the 30% probability level [Symmetry codes: (i) -x + 1, -y + 1, -z + 1; (ii) x, -y + 1/2, z + 1/2; (iii) -x + 2, -y + 1, -z + 1; (iv) x, -y + 1/2, z - 1/2].

**Fig. 2.**
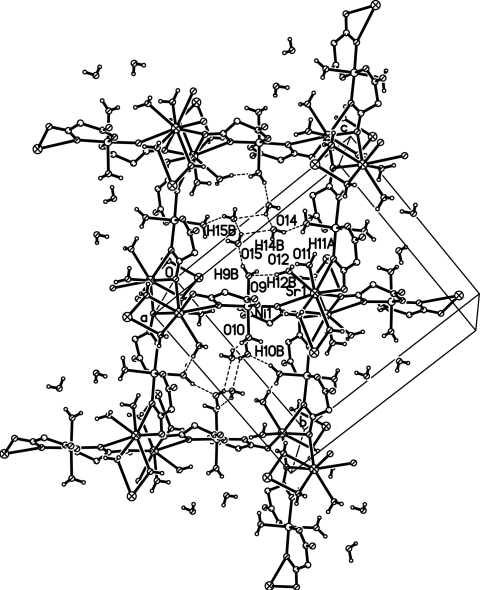
The packing diagram of (I), viewed down the a axis, showing its 20-membered structure and water cluster in the direaction of bc plane.

**Fig. 3.**
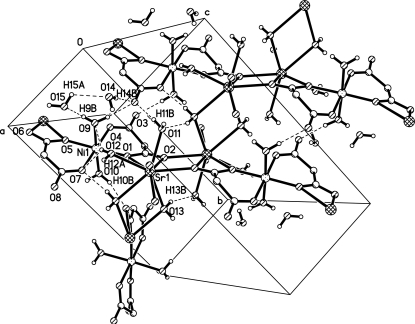
The packing diagram of (I), showing hydrogen-bonding interactions between the [Ni(mal)2(H2O)2]2- dianions and water molecules, viewed down the c axis.

## supplementary crystallographic information

### Comment

Here we report the structure of the title compound, (I), [SrNi(mal)_2_(H_2_O)_5_].2H_2_O (mal = malonate dianion). Although isotypic complex [SrCo(mal)_2_(H_2_O)_5_].2H_2_O (Gil de Muro *et al.*, 1999) had been reported, the difficulty in locating the water hydrogen atoms prevents from description of hydrogen bonding for the structure. Herein, we report the structure of the title heterobimetallic malonate complex, (I), with dianionic [Ni(C_3_H_2_O_4_)_2_(H_2_O)_2_]^2-^ structure,

The asymmetric unit in the structure of (I) comprises one Sr atom, one [Ni(C_3_H_2_O_4_)_2_(H_2_O)_2_]^2-^ dianion, three coordinated water and two solvent water molecules, and is shown in Fig. 1 in a symmetry-expanded view which displays the full coordination of the Ni and Sr atom. Selected geometric parameters are given in Table 1. The Ni atom has a slightly enlongated axial distortion octahedral coordination. The Ni1 atom deviates by 0.0251 (4)Å from the least-squares plane defined by four O atoms subtended by the two ligands at its metal atom. The O—Ni—O angles are close to 90°, the mean Ni1—O bond distance are 2.026 (2) Å. These are somewhat shorter than those (2.051 (3) Å) in [CaNi(mal)_2_(H_2_O)_2_].2H_2_O (Gil de Muro *et al.*, 2000), while the Ni—O_water_ bonds except for Ni1—O9(2.038 (3) Å) are in good agreement with those (2.066 (4) Å) observed for above-mentioned compounds. The variability of the coordination modes of malonate ligands, monodentate, bidentate chelating, chelated six-membered and bridging bonding modes, are all present. As can be seen in Table 1, the O—C—O angles for carboxylate groups of two malonates range from 120.9 (3) to 123.9 (4)°, all of the C—O bond distances are in the range 1.246 (4)–1.265 (4)Å except the shortest O7—C6 being 1.233 (4)Å and the longest O1—C1 being 1.272 (4) Å. These indicate that all of carboxylate groups of malonates are somewhat delocalized.

The coordination polyhedron around the Sr atom is a nine-coordinate distorted tricapped trigonal prism defined by five O atoms from three carboxylate groups and four O atoms from coordinated water molecules. Two of the three carboxylate groups coordinate with the Sr atom in a chelate fashion, whereas the other one is in a bridging mode and serve as bridges between two Sr atoms. Atoms O1, O2, O13 and O13^iii^ (see Fig. 1 for symmetry codes) are coplanar within deviations of less than 0.128 (2)Å and form one uncapped rectangular face. Atoms O2, O13, O5^ii^ and O11 are coplanar, with no deviations of more than 0.150 (2) Å, and form the second rectangular face, with O6^ii^ and O2^i^ as the capping atoms. Atoms O1, O11, O5^ii^ and O13^iii^ are in the same plane with displacements of less than 0.142 (3) Å, forming the third rectangular face capped by atom O12. The angle between the planes defined by the triangles O1/O2/O11 and O5^ii^/O13/O13^iii^ is 2.67 (14)°. The average Sr1—O distance are 2.649 (2) Å, slightly shorter than those in [SrCo(mal)_2_(H_2_O)_5_].2H_2_O (Gil de Muro *et al.*, 1999). The strontium polyhedra are linked to a dimer *via* bridge atoms O2 and O2^i^ as a common edge. The dianionic [Ni(mal)_2_(H_2_O)_2_]^2-^ act as building blocks to coordinating to three Sr atoms (Fig. 1) *via* atoms O5, O6, O1 and O2. As the result, each group of four atoms Ni, and six Sr build up a decanuclear 20-membered ring at *bc* plane direction. These are further joined into a two-dimensional layer (Fig. 2). The Sr dimers are further linked between them along the a direction *via* other common edge, O13—O13^iii^, due to the presence of an inversion center at the middle point of these edges, forming a zigzag SrO_7_ chain. And as chains of edge-sharing Sr polyhedra propagate in the direction of the *a* axis and strontium polyhedra chains are linked between them by corner-sharing NiO_6_ distorted octahedra, thus, three-dimensional metal-organic framework is completed.

Solvent water molecules are embed in such decanuclear 20-membered rings composed of four [Ni(mal)_2_(H_2_O)_2_]^2-^ connecting the Sr dimers. Hydrogen-bonding interactions between them are responsible for the conformation of a R4 water cluster with overhanging water molecules (Infantes & Motherwell, 2002). The detailed structure of the water cluster is shown in Figure 2. First, the solvent water molecules are linked into a D5 water chain of O12, O9, O15, O14 and O10^ii^. Atom H15A as a bifurcated hydrogen one, the four solvent water molecules are further connected *via* H15A and symmetry-expanded hydrogen bonds and produce this R4 water cluster. As can be seen from Table 2 and Figure 2, within the water cluster, water molecules O14 displays tetrahedral geometry with double hydrogen-bond donors and acceptors. The O···O distances are in range of 2.663 (5)–3.094 (5) Å with an average of 2.89 (1) Å.

The dianionic [Ni(mal)_2_(H_2_O)_2_]^2-^ act as both hydrogen-bonded donors and acceptors and engage in distinct hydrogen-bonding interactions (Fig. 3 and Table 2). Except for their conformation of *R*_2_^2^(12) ring between two adjacent dianions, at least there are the following hydrogen-bonded graph sets (Bernstein, *et al.*, 1995): (1) the non-coordinated O8 atom is involved in forming strong hydrogen bond O11—H11B···O8^vii^ and responsible for the conformation of two 8-membered hydrogen bonded ring *R*_3_^3^(8) and *R*_3_^2^(8); (2) hydrogen bond O12—H12B···O9 engage in the formation of a S(6) ring and a three-center hydrogen bond *R*_1_^2^(4) *via* atom O7. (3) H atoms of water molecule O9 act as proton donors, coordinate to O15 and O8^vii^ as acceptors, and further *via* water molecule O14, build up an 8-membered ring *R*_4_^3^(8) motif; (4) hydrogen bond O10—H10B···O6^ix^ participate in the conformation of an 8-membered hydrogen bonded ring *R*_2_^2^(8) and a S(8) hydrogen bonded ring motif *via* two Sr atoms and one Ni atom. In addition, around the Sr dimers there is a S(6) ring hydrogen-bonded graph set *via* O13—H13B···O11^i^ hydrogen bonds. These play an important role in manipulation of the three-dimensional metal-organic framework with pore.

### Experimental

The title complex was prepared under continuous stirring with successive addition of CH_2_(COONa)_2_.H_2_O (0.33 g, 2 mmol), NiCl_2_.6H_2_O (0.24 g, 1 mmol), and Sr(NO_3_)_2_ (0.21 g, 1 mmol) to distilled water (10 ml) at room temperature. After filtration, slow evaporation over a period of two days at room temperature provided pale green prisms of (I).

### Refinement

The H atoms of the water molecule were found in difference Fourier maps. However, during refinement, they were fixed at O–H distances of 0.85 Å and their *U*_iso_ values were set at 1.2 *U*_eq_(O). The H atoms of CH_2_ groups were treated as riding, with C–H = 0.97 Å, and *U*_iso_ (H) = 1.5 *U*_eq_(C).

### Crystal data

**Table:**

[NiSr(C_3_H_2_O_4_)_2_(H_2_O)_5_]·2H_2_O	*F*(000) = 960
*M**_r_* = 476.53	*D*_x_ = 2.142 Mg m^−^^3^
Monoclinic, *P*2_1_/*c*	Mo *K*α radiation, λ = 0.71073 Å
Hall symbol: -P 2ybc	Cell parameters from 11235 reflections
*a* = 6.7745 (14) Å	θ = 1.3–28.2°
*b* = 14.220 (3) Å	µ = 4.97 mm^−^^1^
*c* = 15.629 (3) Å	*T* = 294 K
β = 101.10 (3)°	Prism, green
*V* = 1477.4 (5) Å^3^	0.12 × 0.06 × 0.04 mm
*Z* = 4	

### Data collection

**Table:**

Rigaku Saturn CCD area-detector diffractometer	2609 independent reflections
Radiation source: rotating anode	2235 reflections with *I* > 2σ(*I*)
confocal	*R*_int_ = 0.045
Detector resolution: 28.57 pixels mm^-1^	θ_max_ = 25.0°, θ_min_ = 2.0°
ω scans	*h* = −7→8
Absorption correction: multi-scan (*CrystalClear*; Rigaku/MSC, 2005)	*k* = −13→16
*T*_min_ = 0.548, *T*_max_ = 0.712	*l* = −18→18
9983 measured reflections	

### Refinement

**Table:**

Refinement on *F*^2^	Primary atom site location: structure-invariant direct methods
Least-squares matrix: full	Secondary atom site location: difference Fourier map
*R*[*F*^2^ > 2σ(*F*^2^)] = 0.035	Hydrogen site location: inferred from neighbouring sites
*wR*(*F*^2^) = 0.098	H-atom parameters constrained
*S* = 1.05	*w* = 1/[σ^2^(*F*_o_^2^) + (0.0667*P*)^2^] where *P* = (*F*_o_^2^ + 2*F*_c_^2^)/3
2609 reflections	(Δ/σ)_max_ = 0.001
208 parameters	Δρ_max_ = 0.78 e Å^−^^3^
0 restraints	Δρ_min_ = −0.59 e Å^−^^3^

### Special details

**Table:**

Geometry. All e.s.d.'s (except the e.s.d. in the dihedral angle between two l.s. planes) are estimated using the full covariance matrix. The cell e.s.d.'s are taken into account individually in the estimation of e.s.d.'s in distances, angles and torsion angles; correlations between e.s.d.'s in cell parameters are only used when they are defined by crystal symmetry. An approximate (isotropic) treatment of cell e.s.d.'s is used for estimating e.s.d.'s involving l.s. planes.
Refinement. Refinement of *F*^2^ against ALL reflections. The weighted *R*-factor *wR* and goodness of fit *S* are based on *F*^2^, conventional *R*-factors *R* are based on *F*, with *F* set to zero for negative *F*^2^. The threshold expression of *F*^2^ > σ(*F*^2^) is used only for calculating *R*-factors(gt) *etc*. and is not relevant to the choice of reflections for refinement. *R*-factors based on *F*^2^ are statistically about twice as large as those based on *F*, and *R*- factors based on ALL data will be even larger.

### Fractional atomic coordinates and isotropic or equivalent isotropic displacement parameters (Å^2^)

**Table:**

	*x*	*y*	*z*	*U*_iso_*/*U*_eq_	
Sr1	0.76917 (4)	0.41527 (3)	0.54827 (2)	0.03336 (14)	
Ni1	0.77798 (6)	0.26471 (3)	0.29429 (3)	0.03640 (16)	
O1	0.6901 (3)	0.35987 (19)	0.37645 (16)	0.0383 (6)	
O2	0.4937 (4)	0.46958 (19)	0.41211 (16)	0.0376 (6)	
O3	0.2090 (5)	0.3344 (3)	0.1625 (3)	0.0892 (15)	
O4	0.4994 (4)	0.2690 (2)	0.21955 (17)	0.0426 (6)	
O5	0.8646 (4)	0.16635 (19)	0.21529 (16)	0.0376 (6)	
O6	1.0210 (4)	0.03826 (19)	0.18917 (16)	0.0393 (6)	
O7	1.0539 (4)	0.2606 (2)	0.37197 (18)	0.0466 (7)	
O8	1.3529 (4)	0.2065 (2)	0.4271 (2)	0.0600 (9)	
C1	0.5431 (5)	0.4164 (3)	0.3563 (2)	0.0353 (8)	
C2	0.4297 (6)	0.4246 (3)	0.2641 (3)	0.0430 (9)	
H2A	0.5089	0.4630	0.2320	0.052*	
H2B	0.3057	0.4584	0.2650	0.052*	
C3	0.3765 (5)	0.3345 (3)	0.2136 (3)	0.0468 (10)	
C4	1.0035 (5)	0.1073 (3)	0.2363 (2)	0.0372 (8)	
C5	1.1565 (7)	0.1145 (4)	0.3191 (3)	0.0618 (13)	
H5A	1.2856	0.1000	0.3042	0.074*	
H5B	1.1275	0.0639	0.3565	0.074*	
C6	1.1875 (5)	0.2012 (3)	0.3748 (2)	0.0399 (9)	
O9	0.6818 (5)	0.1619 (2)	0.3674 (2)	0.0703 (11)	
H9A	0.5832	0.1782	0.3899	0.084*	
H9B	0.7235	0.1063	0.3805	0.084*	
O10	0.8706 (3)	0.37394 (19)	0.22446 (16)	0.0380 (6)	
H10A	0.9763	0.3629	0.2042	0.046*	
H10B	0.8794	0.4243	0.2542	0.046*	
O11	0.4547 (3)	0.32253 (19)	0.56368 (16)	0.0384 (6)	
H11A	0.4583	0.2973	0.6130	0.046*	
H11B	0.4168	0.2852	0.5212	0.046*	
O12	0.8968 (4)	0.24515 (19)	0.54373 (17)	0.0418 (6)	
H12A	1.0020	0.2298	0.5801	0.050*	
H12B	0.8940	0.2226	0.4933	0.050*	
O13	0.8855 (4)	0.58384 (18)	0.51241 (16)	0.0380 (6)	
H13A	0.9186	0.6223	0.5544	0.046*	
H13B	0.7751	0.5986	0.4787	0.046*	
O14	0.5916 (6)	0.0732 (3)	0.5637 (2)	0.0772 (11)	
H14A	0.6736	0.1063	0.6009	0.093*	
H14B	0.5234	0.1080	0.5238	0.093*	
O15	0.8248 (5)	0.0017 (3)	0.4433 (2)	0.0769 (11)	
H15A	0.7252	−0.0157	0.4633	0.092*	
H15B	0.8963	−0.0419	0.4283	0.092*	

### Atomic displacement parameters (Å^2^)

**Table:**

	*U*^11^	*U*^22^	*U*^33^	*U*^12^	*U*^13^	*U*^23^
Sr1	0.0315 (2)	0.0324 (2)	0.0327 (2)	0.00063 (12)	−0.00229 (14)	−0.00001 (13)
Ni1	0.0387 (3)	0.0331 (3)	0.0342 (3)	0.0033 (2)	−0.0009 (2)	−0.0006 (2)
O1	0.0380 (13)	0.0386 (16)	0.0344 (13)	0.0057 (11)	−0.0024 (11)	−0.0025 (11)
O2	0.0367 (13)	0.0369 (16)	0.0363 (13)	0.0007 (11)	−0.0004 (11)	−0.0015 (12)
O3	0.0387 (16)	0.121 (4)	0.097 (3)	0.0142 (19)	−0.0150 (17)	−0.071 (3)
O4	0.0397 (14)	0.0407 (16)	0.0439 (15)	−0.0028 (12)	−0.0005 (12)	−0.0084 (12)
O5	0.0378 (13)	0.0351 (16)	0.0369 (13)	0.0038 (11)	−0.0004 (10)	−0.0023 (11)
O6	0.0427 (14)	0.0339 (15)	0.0374 (14)	0.0031 (11)	−0.0015 (11)	−0.0030 (12)
O7	0.0522 (16)	0.0419 (17)	0.0385 (14)	0.0117 (13)	−0.0093 (12)	−0.0049 (12)
O8	0.0359 (14)	0.070 (2)	0.0674 (19)	0.0041 (14)	−0.0073 (14)	−0.0353 (18)
C1	0.0328 (17)	0.033 (2)	0.037 (2)	−0.0026 (15)	0.0001 (15)	0.0012 (16)
C2	0.040 (2)	0.046 (3)	0.039 (2)	0.0068 (17)	−0.0019 (16)	−0.0027 (18)
C3	0.0317 (18)	0.058 (3)	0.047 (2)	0.0000 (18)	−0.0009 (16)	−0.018 (2)
C4	0.0377 (19)	0.034 (2)	0.039 (2)	−0.0009 (16)	0.0039 (16)	−0.0020 (17)
C5	0.051 (2)	0.050 (3)	0.069 (3)	0.017 (2)	−0.026 (2)	−0.023 (2)
C6	0.0361 (19)	0.040 (2)	0.041 (2)	−0.0013 (16)	0.0014 (16)	−0.0027 (17)
O9	0.073 (2)	0.048 (2)	0.102 (3)	0.0248 (17)	0.048 (2)	0.0311 (19)
O10	0.0368 (12)	0.0344 (15)	0.0393 (14)	0.0015 (11)	−0.0011 (11)	−0.0032 (12)
O11	0.0390 (13)	0.0378 (16)	0.0341 (13)	−0.0024 (11)	−0.0039 (10)	0.0006 (11)
O12	0.0435 (14)	0.0413 (17)	0.0358 (13)	0.0036 (12)	−0.0040 (11)	0.0002 (11)
O13	0.0344 (12)	0.0370 (16)	0.0387 (14)	0.0030 (10)	−0.0025 (11)	−0.0025 (11)
O14	0.076 (2)	0.081 (3)	0.065 (2)	−0.016 (2)	−0.0118 (18)	0.0063 (19)
O15	0.080 (2)	0.058 (2)	0.097 (3)	0.025 (2)	0.030 (2)	0.027 (2)

### Geometric parameters (Å, °)

**Table:**

Sr1—O11	2.556 (2)	C1—C2	1.502 (5)
Sr1—O12	2.574 (3)	C2—C3	1.512 (6)
Sr1—O2^i^	2.581 (3)	C2—H2A	0.9700
Sr1—O6^ii^	2.598 (3)	C2—H2B	0.9700
Sr1—O13	2.618 (3)	C4—C5	1.498 (6)
Sr1—O2	2.660 (3)	C5—C6	1.500 (6)
Sr1—O13^iii^	2.688 (2)	C5—H5A	0.9700
Sr1—O1	2.751 (3)	C5—H5B	0.9700
Sr1—O5^ii^	2.816 (3)	O9—H9A	0.8447
Ni1—O4	2.020 (3)	O9—H9B	0.8514
Ni1—O7	2.024 (3)	O10—H10A	0.8512
Ni1—O5	2.026 (2)	O10—H10B	0.8504
Ni1—O1	2.032 (3)	O11—H11A	0.8461
Ni1—O9	2.038 (3)	O11—H11B	0.8497
Ni1—O10	2.064 (3)	O12—H12A	0.8499
O1—C1	1.272 (4)	O12—H12B	0.8484
O2—C1	1.247 (4)	O13—H13A	0.8504
O3—C3	1.255 (5)	O13—H13B	0.8538
O4—C3	1.240 (5)	O14—H14A	0.8626
O5—C4	1.257 (5)	O14—H14B	0.8589
O6—C4	1.247 (5)	O15—H15A	0.8350
O7—C6	1.233 (5)	O15—H15B	0.8470
O8—C6	1.256 (5)		
			
O11—Sr1—O12	78.94 (8)	O4—Ni1—O7	178.51 (10)
O11—Sr1—O2^i^	71.25 (9)	O4—Ni1—O5	90.94 (10)
O12—Sr1—O2^i^	148.61 (8)	O7—Ni1—O5	90.19 (11)
O11—Sr1—O6^ii^	118.33 (8)	O4—Ni1—O1	89.44 (10)
O12—Sr1—O6^ii^	95.34 (8)	O7—Ni1—O1	89.39 (10)
O2^i^—Sr1—O6^ii^	90.35 (8)	O5—Ni1—O1	178.06 (10)
O11—Sr1—O13	141.58 (8)	O4—Ni1—O9	88.97 (13)
O12—Sr1—O13	137.57 (8)	O7—Ni1—O9	90.06 (14)
O2^i^—Sr1—O13	73.77 (8)	O5—Ni1—O9	90.45 (12)
O6^ii^—Sr1—O13	76.88 (8)	O1—Ni1—O9	87.66 (12)
O11—Sr1—O2	75.89 (8)	O4—Ni1—O10	90.96 (11)
O12—Sr1—O2	115.99 (8)	O7—Ni1—O10	89.96 (11)
O2^i^—Sr1—O2	66.27 (9)	O5—Ni1—O10	92.53 (10)
O6^ii^—Sr1—O2	148.12 (9)	O1—Ni1—O10	89.36 (11)
O13—Sr1—O2	75.91 (8)	O9—Ni1—O10	177.02 (12)
O11—Sr1—O13^iii^	146.45 (8)	C1—O1—Ni1	125.2 (2)
O12—Sr1—O13^iii^	71.04 (8)	C1—O1—Sr1	93.2 (2)
O2^i^—Sr1—O13^iii^	140.29 (8)	Ni1—O1—Sr1	141.44 (12)
O6^ii^—Sr1—O13^iii^	79.85 (8)	C1—O2—Sr1^i^	147.4 (2)
O13—Sr1—O13^iii^	66.54 (9)	C1—O2—Sr1	98.2 (2)
O2—Sr1—O13^iii^	103.91 (8)	Sr1^i^—O2—Sr1	113.73 (9)
O11—Sr1—O1	86.27 (8)	C3—O4—Ni1	127.2 (3)
O12—Sr1—O1	72.98 (8)	C4—O5—Ni1	126.3 (2)
O2^i^—Sr1—O1	113.72 (8)	C4—O5—Sr1^iv^	89.6 (2)
O6^ii^—Sr1—O1	150.82 (7)	Ni1—O5—Sr1^iv^	143.89 (12)
O13—Sr1—O1	93.63 (8)	C4—O6—Sr1^iv^	100.3 (2)
O2—Sr1—O1	47.73 (8)	C6—O7—Ni1	128.9 (3)
O13^iii^—Sr1—O1	71.10 (8)	O2—C1—O1	120.9 (3)
O11—Sr1—O5^ii^	75.33 (8)	O2—C1—C2	117.9 (3)
O12—Sr1—O5^ii^	67.66 (8)	O1—C1—C2	121.2 (3)
O2^i^—Sr1—O5^ii^	94.87 (8)	O2—C1—Sr1	58.32 (19)
O6^ii^—Sr1—O5^ii^	47.39 (8)	O1—C1—Sr1	62.55 (19)
O13—Sr1—O5^ii^	123.45 (8)	C2—C1—Sr1	175.7 (3)
O2—Sr1—O5^ii^	149.50 (7)	C1—C2—C3	117.5 (4)
O13^iii^—Sr1—O5^ii^	105.60 (8)	C1—C2—H2A	107.9
O1—Sr1—O5^ii^	138.92 (8)	C3—C2—H2A	107.9
O11—Sr1—C4^ii^	98.18 (9)	C1—C2—H2B	107.9
O12—Sr1—C4^ii^	79.28 (9)	C3—C2—H2B	107.9
O2^i^—Sr1—C4^ii^	95.17 (9)	H2A—C2—H2B	107.2
O6^ii^—Sr1—C4^ii^	23.50 (9)	O4—C3—O3	123.9 (4)
O13—Sr1—C4^ii^	100.31 (9)	O4—C3—C2	120.5 (3)
O2—Sr1—C4^ii^	161.43 (9)	O3—C3—C2	115.4 (4)
O13^iii^—Sr1—C4^ii^	90.77 (9)	O6—C4—O5	121.6 (4)
O1—Sr1—C4^ii^	150.52 (9)	O6—C4—C5	115.8 (3)
O5^ii^—Sr1—C4^ii^	24.11 (9)	O5—C4—C5	122.6 (3)
O11—Sr1—C1	80.11 (9)	O6—C4—Sr1^iv^	56.20 (19)
O12—Sr1—C1	94.85 (9)	O5—C4—Sr1^iv^	66.3 (2)
O2^i^—Sr1—C1	89.63 (9)	C5—C4—Sr1^iv^	167.6 (3)
O6^ii^—Sr1—C1	160.39 (9)	C4—C5—C6	123.6 (4)
O13—Sr1—C1	84.30 (9)	C4—C5—H5A	106.4
O2—Sr1—C1	23.50 (9)	C6—C5—H5A	106.4
O13^iii^—Sr1—C1	87.70 (9)	C4—C5—H5B	106.4
O1—Sr1—C1	24.22 (8)	C6—C5—H5B	106.4
O5^ii^—Sr1—C1	152.04 (9)	H5A—C5—H5B	106.5
C4^ii^—Sr1—C1	174.11 (10)	O7—C6—O8	122.6 (4)
O11—Sr1—Sr1^i^	70.32 (6)	O7—C6—C5	121.5 (3)
O12—Sr1—Sr1^i^	140.14 (6)	O8—C6—C5	115.8 (4)
O2^i^—Sr1—Sr1^i^	33.70 (6)	Ni1—O9—H9A	113.6
O6^ii^—Sr1—Sr1^i^	121.22 (6)	Ni1—O9—H9B	132.4
O13—Sr1—Sr1^i^	71.83 (6)	H9A—O9—H9B	114.0
O2—Sr1—Sr1^i^	32.57 (5)	Ni1—O10—H10A	114.9
O13^iii^—Sr1—Sr1^i^	126.73 (6)	Ni1—O10—H10B	110.0
O1—Sr1—Sr1^i^	80.14 (6)	H10A—O10—H10B	112.5
O5^ii^—Sr1—Sr1^i^	124.90 (5)	Sr1—O11—H11A	115.2
C4^ii^—Sr1—Sr1^i^	128.87 (7)	Sr1—O11—H11B	112.4
C1—Sr1—Sr1^i^	55.97 (7)	H11A—O11—H11B	113.4
O11—Sr1—H13B	125.9	Sr1—O12—H12A	117.7
O12—Sr1—H13B	145.7	Sr1—O12—H12B	115.7
O2^i^—Sr1—H13B	64.5	H12A—O12—H12B	112.9
O6^ii^—Sr1—H13B	92.1	Sr1—O13—Sr1^iii^	113.46 (9)
O13—Sr1—H13B	17.5	Sr1—O13—H13A	118.0
O2—Sr1—H13B	59.0	Sr1^iii^—O13—H13A	98.7
O13^iii^—Sr1—H13B	77.4	Sr1—O13—H13B	95.3
O1—Sr1—H13B	84.4	Sr1^iii^—O13—H13B	120.0
O5^ii^—Sr1—H13B	135.9	H13A—O13—H13B	112.8
C4^ii^—Sr1—H13B	114.9	H14A—O14—H14B	111.1
C1—Sr1—H13B	70.3	H15A—O15—H15B	115.6
Sr1^i^—Sr1—H13B	55.5		
			
O4—Ni1—O1—C1	−22.3 (3)	O1—Ni1—O7—C6	162.7 (3)
O7—Ni1—O1—C1	158.6 (3)	O9—Ni1—O7—C6	75.0 (3)
O9—Ni1—O1—C1	−111.3 (3)	O10—Ni1—O7—C6	−107.9 (3)
O10—Ni1—O1—C1	68.7 (3)	Sr1^i^—O2—C1—O1	−168.7 (3)
O4—Ni1—O1—Sr1	151.6 (2)	Sr1—O2—C1—O1	−0.5 (4)
O7—Ni1—O1—Sr1	−27.5 (2)	Sr1^i^—O2—C1—C2	14.1 (6)
O9—Ni1—O1—Sr1	62.6 (2)	Sr1—O2—C1—C2	−177.7 (3)
O10—Ni1—O1—Sr1	−117.47 (19)	Sr1^i^—O2—C1—Sr1	−168.2 (5)
O11—Sr1—O1—C1	74.0 (2)	Ni1—O1—C1—O2	176.6 (2)
O12—Sr1—O1—C1	153.6 (2)	Sr1—O1—C1—O2	0.4 (4)
O2^i^—Sr1—O1—C1	6.4 (2)	Ni1—O1—C1—C2	−6.3 (5)
O6^ii^—Sr1—O1—C1	−136.8 (2)	Sr1—O1—C1—C2	177.6 (3)
O13—Sr1—O1—C1	−67.4 (2)	Ni1—O1—C1—Sr1	176.2 (3)
O2—Sr1—O1—C1	−0.23 (19)	O11—Sr1—C1—O2	77.3 (2)
O13^iii^—Sr1—O1—C1	−131.1 (2)	O12—Sr1—C1—O2	155.2 (2)
O5^ii^—Sr1—O1—C1	136.6 (2)	O2^i^—Sr1—C1—O2	6.3 (2)
C4^ii^—Sr1—O1—C1	174.1 (2)	O6^ii^—Sr1—C1—O2	−83.7 (3)
Sr1^i^—Sr1—O1—C1	3.4 (2)	O13—Sr1—C1—O2	−67.4 (2)
O11—Sr1—O1—Ni1	−100.9 (2)	O13^iii^—Sr1—C1—O2	−134.1 (2)
O12—Sr1—O1—Ni1	−21.35 (18)	O1—Sr1—C1—O2	−179.6 (4)
O2^i^—Sr1—O1—Ni1	−168.55 (17)	O5^ii^—Sr1—C1—O2	106.0 (3)
O6^ii^—Sr1—O1—Ni1	48.3 (3)	Sr1^i^—Sr1—C1—O2	4.47 (18)
O13—Sr1—O1—Ni1	117.59 (19)	O11—Sr1—C1—O1	−103.1 (2)
O2—Sr1—O1—Ni1	−175.2 (2)	O12—Sr1—C1—O1	−25.2 (2)
O13^iii^—Sr1—O1—Ni1	53.91 (18)	O2^i^—Sr1—C1—O1	−174.1 (2)
O5^ii^—Sr1—O1—Ni1	−38.4 (2)	O6^ii^—Sr1—C1—O1	95.9 (3)
C4^ii^—Sr1—O1—Ni1	−0.9 (3)	O13—Sr1—C1—O1	112.1 (2)
C1—Sr1—O1—Ni1	−175.0 (4)	O2—Sr1—C1—O1	179.6 (4)
Sr1^i^—Sr1—O1—Ni1	−171.6 (2)	O13^iii^—Sr1—C1—O1	45.5 (2)
O11—Sr1—O2—C1	−97.7 (2)	O5^ii^—Sr1—C1—O1	−74.4 (3)
O12—Sr1—O2—C1	−27.7 (2)	Sr1^i^—Sr1—C1—O1	−176.0 (2)
O2^i^—Sr1—O2—C1	−173.1 (3)	O2—C1—C2—C3	−139.2 (4)
O6^ii^—Sr1—O2—C1	140.8 (2)	O1—C1—C2—C3	43.6 (5)
O13—Sr1—O2—C1	108.7 (2)	Ni1—O4—C3—O3	176.4 (4)
O13^iii^—Sr1—O2—C1	47.7 (2)	Ni1—O4—C3—C2	1.4 (6)
O1—Sr1—O2—C1	0.2 (2)	C1—C2—C3—O4	−41.0 (5)
O5^ii^—Sr1—O2—C1	−117.4 (2)	C1—C2—C3—O3	143.6 (4)
C4^ii^—Sr1—O2—C1	−171.0 (3)	Sr1^iv^—O6—C4—O5	11.5 (4)
Sr1^i^—Sr1—O2—C1	−173.1 (3)	Sr1^iv^—O6—C4—C5	−169.0 (3)
O11—Sr1—O2—Sr1^i^	75.43 (11)	Ni1—O5—C4—O6	165.8 (3)
O12—Sr1—O2—Sr1^i^	145.39 (9)	Sr1^iv^—O5—C4—O6	−10.5 (4)
O2^i^—Sr1—O2—Sr1^i^	0.0	Ni1—O5—C4—C5	−13.6 (5)
O6^ii^—Sr1—O2—Sr1^i^	−46.06 (17)	Sr1^iv^—O5—C4—C5	170.2 (4)
O13—Sr1—O2—Sr1^i^	−78.20 (11)	Ni1—O5—C4—Sr1^iv^	176.2 (3)
O13^iii^—Sr1—O2—Sr1^i^	−139.18 (9)	O6—C4—C5—C6	166.9 (4)
O1—Sr1—O2—Sr1^i^	173.35 (16)	O5—C4—C5—C6	−13.7 (7)
O5^ii^—Sr1—O2—Sr1^i^	55.7 (2)	Sr1^iv^—C4—C5—C6	119.5 (12)
C4^ii^—Sr1—O2—Sr1^i^	2.1 (3)	Ni1—O7—C6—O8	−179.3 (3)
C1—Sr1—O2—Sr1^i^	173.1 (3)	Ni1—O7—C6—C5	−3.2 (6)
O5—Ni1—O4—C3	−156.7 (3)	C4—C5—C6—O7	22.8 (7)
O1—Ni1—O4—C3	25.2 (3)	C4—C5—C6—O8	−160.8 (4)
O9—Ni1—O4—C3	112.8 (3)	O11—Sr1—O13—Sr1^iii^	−156.16 (10)
O10—Ni1—O4—C3	−64.2 (3)	O12—Sr1—O13—Sr1^iii^	0.99 (17)
O4—Ni1—O5—C4	−155.1 (3)	O2^i^—Sr1—O13—Sr1^iii^	178.76 (12)
O7—Ni1—O5—C4	23.9 (3)	O6^ii^—Sr1—O13—Sr1^iii^	84.49 (10)
O9—Ni1—O5—C4	−66.1 (3)	O2—Sr1—O13—Sr1^iii^	−112.28 (11)
O10—Ni1—O5—C4	113.9 (3)	O13^iii^—Sr1—O13—Sr1^iii^	0.0
O4—Ni1—O5—Sr1^iv^	18.5 (2)	O1—Sr1—O13—Sr1^iii^	−67.58 (10)
O7—Ni1—O5—Sr1^iv^	−162.5 (2)	O5^ii^—Sr1—O13—Sr1^iii^	93.71 (11)
O9—Ni1—O5—Sr1^iv^	107.5 (2)	C4^ii^—Sr1—O13—Sr1^iii^	86.33 (11)
O10—Ni1—O5—Sr1^iv^	−72.5 (2)	C1—Sr1—O13—Sr1^iii^	−89.97 (11)
O5—Ni1—O7—C6	−15.4 (3)	Sr1^i^—Sr1—O13—Sr1^iii^	−145.97 (10)

Symmetry codes: (i) −*x*+1, −*y*+1, −*z*+1; (ii) *x*, −*y*+1/2, *z*+1/2; (iii) −*x*+2, −*y*+1, −*z*+1; (iv) *x*, −*y*+1/2, *z*−1/2.

### Hydrogen-bond geometry (Å, °)

**Table:**

*D*—H···*A*	*D*—H	H···*A*	*D*···*A*	*D*—H···*A*
O15—H15A···O14^v^	0.84	2.26	2.997 (5)	148
O15—H15A···O14	0.84	2.33	2.867 (5)	123
O15—H15B···O3^vi^	0.85	2.29	2.881 (7)	127
O14—H14B···O8^vii^	0.86	2.21	3.072 (5)	176
O14—H14A···O10^ii^	0.86	2.14	2.936 (4)	152
O13—H13B···O11^i^	0.85	1.93	2.728 (4)	155
O13—H13A···O7^iii^	0.85	2.01	2.836 (4)	163
O12—H12B···O7	0.85	2.42	3.080 (4)	135
O12—H12B···O9	0.85	2.36	3.094 (5)	144
O12—H12A···O3^viii^	0.85	1.94	2.772 (4)	166
O11—H11B···O8^vii^	0.85	1.83	2.681 (4)	176
O11—H11A···O4^ii^	0.85	1.89	2.727 (4)	172
O10—H10B···O6^ix^	0.85	1.91	2.728 (4)	162
O10—H10A···O3^x^	0.85	1.86	2.714 (4)	178
O9—H9B···O15	0.85	1.84	2.663 (5)	162
O9—H9A···O8^vii^	0.84	1.81	2.652 (4)	173

Symmetry codes: (v) −*x*+1, −*y*, −*z*+1; (vi) −*x*+1, *y*−1/2, −*z*+1/2; (vii) *x*−1, *y*, *z*; (ii) *x*, −*y*+1/2, *z*+1/2; (i) −*x*+1, −*y*+1, −*z*+1; (iii) −*x*+2, −*y*+1, −*z*+1; (viii) *x*+1, −*y*+1/2, *z*+1/2; (ix) −*x*+2, *y*+1/2, −*z*+1/2; (x) *x*+1, *y*, *z*.
